# Silenced by violence: mental health impact of Colombia’s armed conflict on women

**DOI:** 10.1192/bji.2025.10058

**Published:** 2026-05

**Authors:** Valentina Belalcazar-Vivas

**Affiliations:** Department of Medicine, University of Pamplona, Colombia.

## Abstract

Colombia’s armed conflict has deeply impacted women’s mental health, particularly in rural and marginalised areas, where violence has become normalised. Despite legal recognition of mental health as a right, access to care remains limited due to geographic, cultural, systemic and security barriers. Mental health programmes lack continuity and funding, while professional training, availability and research in trauma-informed care are still insufficient. Institutional mistrust and ongoing threats further silence women’s suffering. This paper describes the current state of mental health services for women affected by armed conflict in Colombia and reflects on the persistent challenges.

Colombia’s internal armed conflict has lasted more than 6 decades, leaving over 10 million victims in its wake, with women accounting for more than 50% of those affected.^1^ Despite the 2016 peace agreement signed with guerrilla groups, violence continues, particularly in departments such as Cauca, Chocó, Arauca, Nariño, Norte de Santander, Putumayo and Valle del Cauca.^2^ Women, particularly those in rural, Indigenous and Afro-Colombian communities, have been disproportionately affected. They face not only the direct consequences of war but also intersecting challenges such as displacement, poverty, gender-based violence and structural neglect.

Although Law 1616 of 2013 established mental health as a fundamental right and mandated integrated care,^3^ implementation remains inconsistent and underfunded. Programmes such as the Psychosocial and Comprehensive Health Program for Victims (PAPSIVI) were created to address the needs of survivors using gender-sensitive and community-based approaches, but they often fail to reach the most affected populations. In this complex context, the psychological consequences of conflict remain largely unaddressed, especially for women in marginalised regions.

## How the normalisation of violence silences women’s suffering

Chronic exposure to conflict and displacement has led to a normalisation of violence. Women are not only affected by the direct consequences of war, but also by the compounding effects of poverty, gender-based violence and long-standing structural neglect. This desensitisation has eroded social support systems and silenced survivors. As a result, many women remain in a state of never-ending trauma, experiencing mood disorders, post-traumatic stress, anxiety and enduring feelings of hatred and vengeance.^4^ In municipalities such as Alto Baudó, Chocó, Indigenous women account for 90% of suicide attempts. In these communities, there isn’t even a specific word for ‘suicide’, nor do people know how to report such events to appropriate authorities.^5^

## Mental health policies for victims of armed conflict

Law 1616 of 2013, known as the ‘mental health law’, recognises mental health as a fundamental right and mandates the provision of integrated care.^3^ However, the implementation of the PAPSIVI has been inconsistent. The programme incorporates gender, ethnic and territorial approaches to address the physical and mental harm suffered by survivors of the armed conflict, but its reach remains limited due to insufficient funding and a lack of continuity. Therefore, although the Ministry of Health reported that 94% of the victims were affiliated with the health system,^6^ this does not necessarily translate into access to adequate psychological or psychiatric care.

## Services available for vulnerable women

In urban areas, particularly in capital cities, women may access both public and private mental health services. However, most victims of the armed conflict live in rural regions, where access is severely limited. For example, in Alto Baudó, Chocó, only 2 psychologists are available to serve approximately 30 000 inhabitants dispersed across 1500 km^2^.^5^ This municipality is located 4 h by road and 2 h by river from Quibdo, the departmental capital, where the only secondary care hospital is located. Many healthcare professionals have resigned from this facility due to delays in payment.

Beyond logistical barriers, cultural and systemic obstacles such as stigma, mistrust in institutions and limited awareness of mental health rights further restrict access to care. Despite government efforts to extend mental health services to women residing in conflict-affected areas, they are unable to benefit from it.

In El Plateado, Cauca, some women have reported needing to request permission from guerilla groups to attend medical appointments in the departmental capital. In some cases, they have been required to present medical documentation to justify their visits and have expressed fear of being punished if the reason for seeking care is not considered acceptable by these groups. This dangerous dynamic not only discourages help-seeking but may also put their lives at risk. In other municipalities, reports of forced recruitment of children into guerilla ranks^7^ further contribute to the fear environment and deters women from approaching mental health services.

In this context, the most frequent request among women is for protection. When personal safety is under threat, mental health often becomes neglected.

## Professional training

Most psychology and psychiatry programmes are based in urban areas. Despite Colombia’s conflict-affected context, training in complex trauma, community-based approaches and mental health care for victims of the armed violence remains limited. In 2022, the Ministry of Health, in collaboration with the International Organization for Migration and public universities, introduced a short-term training programme for local providers as part of efforts to deploy more mental health professionals to territories historically impacted by conflict.^8^ However, due to security threats, including the interception of ambulances and targeted killings of physicians and health care workers,^9^ many professionals are hesitant to relocate to rural areas, where Indigenous and Afro-Colombian women continue to experience systemic neglect.

## Research

Several studies have documented the psychological consequences of the conflict in the population’s mental health. Research from University of the Andes showed that displaced individuals experience higher rates of depression, anxiety, suicidal ideation and psychosomatic symptoms.^10^ A recent review of 140 articles found that the most prevalent disorders among victims are depression, anxiety and post-traumatic stress disorder.^11^ However, the review also highlighted significant limitations in research, noting that most studies are descriptive and lack methodological rigor. This underscores the urgent need for more comprehensive, international and gender-sensitive research to create targeted mental health interventions for women affected by armed conflict.

## Current challenges

Colombia faces major challenges in responding to the mental health needs of women affected by armed conflict. One critical issue is limited coverage. Despite legal advances, mental health services remain scarce in remote areas. This lack of availability, combined with logistical and security barriers, leaves thousands of women without access to essential psychological support.

Another challenge is the cultural mismatch between western clinical models and the worldviews of Indigenous and Afro-Colombian communities. Many women do not recognise their distress as a medical condition, as violence is widely normalised in the population, or may prefer traditional healing methods to avoid alerting the guerilla groups.

Institutional distrust plays a key role. Due to past experiences of revictimisation, bureaucratic hurdles and ongoing violence, many women are hesitant to engage with state services. This mistrust is particularly strong in territories where armed groups still exert control and where navigating health systems may require dangerous negotiations.

The discontinuous funding and political instability threaten the sustainability of psychosocial programmes. Many initiatives rely heavily on international cooperation and lack long-term government investment. This not only weakens the impact of current efforts but also prevents the development of robust, community-rooted mental health systems.

## Future approaches

To address these challenges, it is essential to ensure sustainable public funding for PAPSIVI and other psychosocial programmes, while expanding training in trauma, gender and intercultural competencies for mental health professionals. Specific protocols should be developed for women exposed to chronic violence, and community participation must be promoted in the design and delivery of mental health services. National security should be strengthened to ensure the safety of the population and mental health professionals.

Despite the urgent need, there is a critical lack of peer-reviewed, high-quality research on the mental health consequences of armed conflict for women in Colombia. Many available studies are descriptive or exploratory, and much of the knowledge on this topic remains in undergraduate and graduate theses housed in university repositories, which are not widely accessible or disseminated. This scarcity of published evidence limits visibility and hinders the development of targeted interventions. Strengthening local research capacity and supporting participatory, gender-sensitive and community-based studies are essential to produce actionable findings. Investment in longitudinal and interdisciplinary research would help ensure that mental health policies reflect the lived experiences of women survivors and respond to their specific needs.
